# The impact of mental health stigma in a young Malaysian lady with recurrent suicidal ideations and moribund presentations to the Emergency Department: a case report

**DOI:** 10.3389/fpsyt.2023.1243015

**Published:** 2023-10-23

**Authors:** Chiara Francine Petrus, Hajar Mohd Salleh Sahimi, Marhani Midin, Jane Tze Yn Lim

**Affiliations:** ^1^Department of Psychiatry, Hospital Canselor Tuanku Muhriz, Kuala Lumpur, Malaysia; ^2^Department of Psychiatry, Faculty of Medicine, University Kebangsaan Malaysia, Kuala Lumpur, Malaysia

**Keywords:** mental health, stigma, suicide-related behavior, emergency department, suicide

## Abstract

**Introduction:**

Mental health stigma (MHS) has been a pervasive social issue and a significant barrier to treatment seeking behavior. The treatment pathways and outcomes for people with mental illness, specifically those with recurrent suicidal ideations and attempts have been influenced by how MHS was experienced in clinical practice.

**Methods:**

We reported a case of a young lady diagnosed with bipolar II disorder, obsessive-compulsive disorder and borderline personality disorder who had recurrent visits to various emergency departments (ED) of tertiary hospitals in Malaysia for suicidality; each time presenting with increased mortality risk and escalating near-lethal outcomes. Among the multiple ED visits after her alleged overdoses of psychotropic medications, thrice she was near-unconscious and had to be intubated for airway protection, subsequently requiring ventilatory support and ICU care. These near-lethal presentations in ED were due to her delays in seeking treatment for fear of re-experiencing the stigmatizing environment among healthcare staff and professionals in the ED.

**Discussion:**

The impact of MHS is detrimental. Effective interventions at various levels in the clinical setting is of utmost importance to prevent the negative consequences of suicidality against MHS.

## Introduction

Mental health stigma (MHS) has been defined as a state of disrepute, disapproval and discrediting of individuals with mental health issues (1). There are many types of stigma; self-stigma, public, professional, family and institutional stigma (1). In a health care setting, the emergency department (ED) is more often than not the first line of contact for patients with mental health issues. Besides mood disorders, psychotic illnesses and substance or alcohol use disorder, suicide and suicide related behaviors (SRB) are common presentations seen in the ED (2, 3). Unfortunately, literature shows that professional and societal stigma are widely present toward these individuals in this environment (4).

Suicide occurs every 40 s, with a reported 800,000 people who died by suicide worldwide every year (5). Specifically in bipolar disorder, 20% of patients committed suicide and 25–50% of them have had suicidal attempts during the course of illness, whereas in borderline personality disorder, at least three quarter of patients attempted suicide with 10% of them completing the act (6). Aside from mental health illnesses, other medical health disorders carry their own risk of suicide. Studies have shown increased suicide attempts as much as seven times among those suffering from polycystic ovarian syndrome (PCOS) compared to controls (7). In some of these cases, there has been a need to escalate treatment to electroconvulsive therapy in order to help with the suicidal ideations (8). Among the pathophysiological processes associated with SRB are hyperactivity of the hypothalamic–pituitary–adrenal (HPA) axis as well as higher mean concentrations of inflammatory mediators found both in the periphery and brain that is accompanied by a dysregulation of the kynurenine pathway (KP) that result in an imbalance of neuroactive metabolites resulting in SRB (9, 10).

Bipolar mood disorder is described as an episodic mood disorder known to have among the highest risk of suicide, whether attempted or completed (11). Commonly in Bipolar II Disorder, the dysphoric and depressive phases of bipolar as seen in our patient, combined with the impulsivity and hopelessness that is also present in borderline personality disorder, further increase the risk for suicidal behaviors (12–14). Utilization of emergency healthcare services by patients with bipolar mood disorder and borderline personality disorder are among the highest (15–17), with suicidal ideations being the most common psychiatric reasons for presentation (18).

Reviewed literature has consistently highlighted the adverse effects of mental health stigma toward these individuals. This stigma forms a barrier in delivering optimal care to break the cycle of recurring visits, with increased burden on the healthcare system. Among the contributing factors are the lack of understanding of mental health issues, fear of rejection and discrimination, limited social support as well as negative encounters with healthcare workers (4, 19). We hypothesized that MHS experienced in front-line setting such as the ED has an impact on the narrative of help-seeking behavior in people with recurrent SRB. This case report highlights the consequences of professional and perceived stigma as well as how extreme and dangerous its impact can be. We report the case of a patient with complex diagnosis of bipolar II disorder, borderline personality disorder (BPD) and obsessive-compulsive disorder (OCD), who had recurrent suicidal thoughts and attempts requiring frequent visits to emergency department (ED). Informed consent was obtained for this case report.

## Case description

Miss E is a 23-year-old ethnic Malay, single female student who is currently studying for a bachelor’s degree in counseling and guidance. She has had congenital right eye blindness and is also on gynecology follow up for polycystic ovarian syndrome (PCOS). She has been diagnosed with Bipolar II Disorder and BPD (fulfilling DSM-5 criteria) since the age of 17 and has been under regular follow up since adolescent years from the Child and Adolescent Psychiatry Clinic before her care was transferred to Adult Psychiatry Clinic after she turned 18 years old.

Miss E has had recurrent hypomanic and depressive episodes since the age of 12. Her hypomanic episodes were approximately twice a year, however, more frequently occurring were her depressive episodes where she would experience low mood with suicidal thoughts, occasional psychomotor agitation and feeling worthless, also in the face of her struggles for ongoing emotional dysregulation and affective instability which would center around the fear of being abandoned by her boyfriend and friends in the background of her chronic feelings of emptiness. Since October 2022, she identified emerging persistent, recurrent, unwanted and independent suicidal thoughts despite in remission of depressive episodes or beyond the recurrent self-harm that she engaged with in response to her emotional dysregulation. She was treated for (OCD) to manage her distressing compulsions to act on the suicidal thoughts.

**Figure 1:**
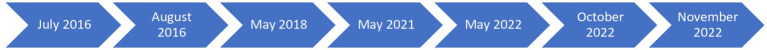
Number of presentations to our ED for suicide related behaviors.

In the course of Miss E’s illness, she required multiple hospitalizations between the years 2016, 2018, 2021 and 2022 due to high suicidal risk or accidental death as a consequence of her self-harm behavior as depicted in Figure 1. Her SRB involved multiple drug ingestions; most commonly psychotropic medications prescribed for treating her psychiatric illness. With each recurrent visit, the severity of her presentation increased; (1) by a more lethal method (amount of ingestion and number of types of medications ingested) or (2) delaying the time between her attempts and presentations to the hospital (crisis to help time). Across these presentations to ED over the years from 2016 to 2022, her mortality risks were evidently increased, with the required level of resuscitation in ED and intensive medical care admission needed to revive her state. In October 2022, she presented in a state of coma, requiring prompt intubation in ED followed by ventilatory support and further stabilization in the intensive care unit.

In Miss E’s recollection of personal experiences in ED, she acknowledged her apprehension in presenting to ED despite the awareness of her medical and psychiatry needs. She reported feeling stigmatized for her recurrent suicidality and attempts, yet futile and helpless in coping with her psychiatric issues especially during emotional crisis that warranted an ED visit. This apprehension would further evoke yet more suicidal thoughts and urges to get rid of such challenging situations in a vicious cycle that could span for 2–3 days before she eventually arrived in ED in moribund state.

## Discussion

In a healthcare facility that provides mental health services, the emergency department is no doubt the first point of access to the mental health crisis. Unfortunately, the care in ED has been in the past, and still is, at some point, being described as hostile, uncaring and counter therapeutic, especially so for those who find their visits appropriate and unavoidable despite being stigmatized by hospital personnel (20–22). SRB, including self-harm, presents risks of accidental death and serious morbidity. Our case description brings this evidence to attention, highlighting the detrimental effect of MHS where help seeking and life-saving intervention were delayed when patients continued to feel misunderstood and SRB wrongly implied by past experiences in ED as an illegitimate reason for attending care. Nevertheless, this case is not an isolated case (23). Numerous research has been done on the rates of stigma associated with suicidality among emergency services professionals who are first responders to mental health care. A study on 156 individuals with a lifetime history of SRB revealed that the highest rate of perceived stigma comes from non-mental health providers with 56.6% from ED personnel (24). They frequently have negative attitudes toward patients who self-harm, which is consistent with the experience encountered by these groups of patients (22). A qualitative analysis also showed that the negative consequences of MHS led to participants’ avoidance of ED, the feelings of shame and unworthiness when seeking immediate care in ED and how these feelings contributed to perpetuating cycle of shame, avoidance and further self-harm when the care in ED was perceived to be punitive (23).

In addressing MHS in ED, multiple factors were at play. Stigma against suicide was linked to emergency nurses’ lack of confidence in handling suicidal patients as well as their lack of understanding of the risk factors and warning signals connected with suicide (25). In addition, stakeholders such as ED clinical leaders including nursing directors, medical directors and behavioral health managers shared a common concern about feeling ill-equipped in managing the self-harm population of interest (26). The unideal situation in ED to manage such SRB and the lack of emotional skills led to clinical uncertainty of disposition for further care after ED visit (26). Similarly in Malaysia, the ED houses multiple cases per day from all disciplines with inadequate space to accommodate each individual’s needs. Adaptations of physical spaces in ED is essential to provide effective care non-judgmentally to these groups of patients with emotional needs. Further to this, the work demands in ED locally require every staff to attend to multiple cases at a time from various disciplines. Stigmatizing behavior also stems from the result of feeling demoralized and powerless from recurring visits of the same patient, with some patients attending ED multiple times a year. Overtime, ED practitioners felt helpless by patients’ attending and found it hard to feel empathy (27).

Research has found that education and staff supervision had positive effects on practitioners’ attitude toward SRB as well as improving patients’ satisfaction (28, 29). Hence, training and regular supervision for practitioners is needed as a way to overcome stigmatizing attitudes toward SRB, particularly those who assess the majority of patients presenting to ED with SRB. To date, there is no standard model of training for such need (22). Locally, efforts to adopt the suicide gatekeeper training for ED practitioners is still ongoing. The important elements in the training such as those from the Attempted Suicide Short Intervention Program that emphasizes human connection can be incorporated (30). Qualitative studies and reports have included the therapeutic values of respect, validation and trust in attending to ED visits by these patients (21, 31).

MHS in ED may represent just the tip of the iceberg. The unmet needs of patients with recurring SRB to ED may reflect the significant barriers of accessing support in the community, social network or professional health system, for which managing stigma at the institutional level including the review of guidelines, protocols, and policy by stakeholders is equally important (27, 32, 33). While efforts to decriminalize suicide have begun locally, these efforts lie not only within the general population but also among the professionals, in this case, ED practitioners. In line with the importance of suicide screening in ED being given a top priority, the gap for patients’ service improvement may stem from access to mental health specialists care in ED (34). Efforts to extend specialized mental health services in ED can be looked into, such as having a team of mental health providers in ED or special task force on suicidality with close liaison and regular multidisciplinary discussions pertaining to recurrent visits in ED for SRB.

In the local context, Malaysia is a multicultural and multireligious nation in which discussions on suicide is a delicate matter due to the associated social, religious and cultural beliefs present. Every religion may have distinct principles and viewpoints about suicide. In 2020, 63.5% of the Malaysian population are Islam by religion, with the second largest religion being Buddhism (18.7%) followed by Christianity (9.1%), Hinduism (6.1%), and other religious groups (9%) (35). Islam, for instance, primarily discourages suicide because it violates the notion of the sanctity of life (36). Buddhism places substantial importance on cultivating respect for life, including human life and discourages actions resulting in dukkha meaning suffering, including any harm to oneself (37). Christianity views human life as the sovereignty of God who is their Creator, raising the debate whether suicide is an unforgivable sin (38). In the cultural aspect, the Malay, Chinese, Indian or indigenous communities, each have their distinctive cultural customs and values which may have an impact on how these communities feel about suicide (39, 40). There is strong stigma attached to those who experience mental health issues, suicide included, within these communities (41). These stigmas also extend toward their family members as being considered shameful to the point of affecting their potential in finding marriageable partners (41). Hence, lowering the negative effects of stigma on suicide in a religious and culturally rich society, even at the point of contact in Emergency Department is also a foundational piece of solution to engage the population at risk of suicide empathetically. A more compassionate atmosphere for people who contemplate suicide can be nurtured through enabling open communication, decreasing stigma, and advancing awareness of mental health issues.

The limitation of this case study is that it represents only a sample of population. Moreover, suicide and SRB are complex phenomenon with many different components interacting over time. In our case, the long-standing comorbid psychiatric disorder starting at an early age and thereafter into adulthood, with recurrent SRB despite the continued treatment of psychotropics also speaks of multiple factors involved in the optimum care of a patient. It is therefore challenging to solely attribute the deterrent in timely presentation for help in ED to only the level of stigma and avoidance of primary healthcare providers. In view of limited resources, this case also did not utilize any questionnaire or self-stigma instruments such as the Internalized Stigma of Mental Illness Scale (ISMI) or the Self-Stigma of Mental Illness Scale (SSMIS) which could add more value to the objective measurement of stigma (42, 43).

## Conclusion

The implication from this study shows the detrimental effect of MHS toward mental health avoidance behavior, which may result in mortality and death by suicide. In the face of MHS, ED must rise above the challenge to meet the complex physical and psychiatric needs of people with recurrent SRB. ED is a crucial part of the healthcare system for people in crisis with potential life-saving interventions. Compassionate care beyond MHS can foster therapeutic interactions. While the work on reducing stigma has been ongoing in many areas, the focus specifically for first responders in ED is still limited. Various advocacy work or mental health awareness and advancing program including workshop, seminar or conference addressing stigma at each level of healthcare providers is important, starting from first responders in ED. Future studies can look into the effectiveness of such interventions with the aim to generalize an evidence-based practice and work across hospitals nationwide.

## Data availability statement

The original contributions presented in the study are included in the article/supplementary material, further inquiries can be directed to the corresponding author.

## Ethics statement

Ethical approval was not required for the studies involving humans because written approval by non-vulnerable study participant acquired. The studies were conducted in accordance with the local legislation and institutional requirements. The participants provided their written informed consent to participate in this study. Written informed consent was obtained from the individual for the publication of any potentially identifiable images or date included in this article. Written informed consent was obtained from the participant/patient(s) for the publication of this case report.

## Author contributions

CP wrote the initial draft of the manuscript that was later revised together with JL. HM, JL, and MM provided regular psychiatric treatment. MM provided expertise and advice. All authors contributed to the article and approved the submitted version.

## References

[1] Subu MA Wati DF Netrida N Priscilla V Dias JM Abraham MS. Types of stigma experienced by patients with mental illness and mental health nurses in Indonesia: a qualitative content analysis. Int J Ment Heal Syst. (2021) 15:1–12. doi: 10.1186/s13033-021-00502-x PMC8524985 34663399

[2] Wise-Harris D Pauly D Kahan D Tan de Bibiana J Hwang SW Stergiopoulos V. “Hospital was the only option”: experiences of frequent emergency department users in mental health. Adm Policy Ment Health Ment Health Serv Res. (2017) 44:405–12. doi: 10.1007/s10488-016-0728-3 26961781

[3] Ceniti AK Heinecke N McInerney SJ. Examining suicide-related presentations to the emergency department. Gen Hosp Psychiatry. (2020) 63:152–7. doi: 10.1016/j.genhosppsych.2018.09.006, PMID: 30268506

[4] van Nieuwenhuizen A Henderson C Kassam A Graham T Murray J Howard LM. Emergency department staff views and experiences on diagnostic overshadowing related to people with mental illness. Epidemiol Psychiatr Sci. (2013) 22:255–62. doi: 10.1017/S2045796012000571, PMID: 23089191 PMC8367326

[5] Ritchie EC. Suicide and the United States army: perspectives from the former psychiatry consultant to the army surgeon general. Cerebrum. (2012) 2012:1. 23447787 PMC3574805

[6] Black DW Blum N Pfohl B Hale N. Suicidal behavior in borderline personality disorder: prevalence, risk factors, prediction, and prevention. J Personal Disord. (2004) 18:226–39. doi: 10.1521/pedi.18.3.226.35445 15237043

[7] Månsson M Holte J Landin-Wilhelmsen K Dahlgren E Johansson A Landén M. Women with polycystic ovary syndrome are often depressed or anxious—a case control study. Psychoneuroendocrinology. (2008) 33:1132–8. doi: 10.1016/j.psyneuen.2008.06.003 18672334

[8] Ni HC Liu HM Tseng MM. Electroconvulsive therapy of a depressed patient with septo-optic dysplasia. J Neuropsychiatry Clin Neurosci. (2008) 20:242–3. doi: 10.1176/jnp.2008.20.2.242, PMID: 18451203

[9] Berardelli I Serafini G Cortese N Fiaschè F O’Connor RC Pompili M. The involvement of hypothalamus–pituitary–adrenal (HPA) axis in suicide risk. Brain Sci. (2020) 10:653. doi: 10.3390/brainsci10090653, PMID: 32967089 PMC7565104

[10] Serafini G Adavastro G Canepa G Capobianco L Conigliaro C Pittaluga F. Abnormalities in kynurenine pathway metabolism in treatment-resistant depression and suicidality: a systematic review. CNS Neurol Disord Drug Targets. (2017) 16:440–53. doi: 10.2174/1871527316666170413110605 28412922

[11] Cassidy F. Risk factors of attempted suicide in bipolar disorder. Suicide Life Threat Behav. (2011) 41:6–11. doi: 10.1111/j.1943-278X.2010.00007.x 21309819

[12] Tondo L Isacsson G Baldessarini RJ. Suicidal behaviour in bipolar disorder: risk and prevention. CNS Drugs. (2003) 17:491–511. doi: 10.2165/00023210-200317070-00003 12751919

[13] Swann AC Dougherty DM Pazzaglia PJ Pham M Steinberg JL Moeller FG. Increased impulsivity associated with severity of suicide attempt history in patients with bipolar disorder. Am J Psychiatr. (2005) 162:1680–7. doi: 10.1176/appi.ajp.162.9.1680, PMID: 16135628

[14] Muhtadie L Johnson SL Carver CS Gotlib IH Ketter TA. A profile approach to impulsivity in bipolar disorder: the key role of strong emotions. Acta Psychiatr Scand. (2014) 129:100–8. doi: 10.1111/acps.12136, PMID: 23600731 PMC4346162

[15] Fleury M-J Rochette L Grenier G Huỳnh C Vasiliadis HM Pelletier É. Factors associated with emergency department use for mental health reasons among low, moderate and high users. Gen Hosp Psychiatry. (2019) 60:111–9. doi: 10.1016/j.genhosppsych.2019.07.006, PMID: 31404825

[16] Aagaard J Aagaard A Buus N. Predictors of frequent visits to a psychiatric emergency room: a large-scale register study combined with a small-scale interview study. Int J Nurs Stud. (2014) 51:1003–13. doi: 10.1016/j.ijnurstu.2013.11.002, PMID: 24315543

[17] Shaikh U Qamar I Jafry F Hassan M Shagufta S Odhejo YI. Patients with borderline personality disorder in emergency departments. Front Psych. (2017) 8:136. doi: 10.3389/fpsyt.2017.00136, PMID: 28824467 PMC5543278

[18] Eseaton PO Oladunjoye AF Anugwom G Onyeaka H Edigin E Osiezagha K. Emergency department utilization by patients with bipolar disorder: a national population-based study. J Affect Disord. (2022) 313:232–4. doi: 10.1016/j.jad.2022.06.086, PMID: 35779671

[19] Knaak S Mantler E Szeto A. Mental illness-related stigma in healthcare: barriers to access and care and evidence-based solutions. Healthc Manage Forum. (2017) 30:111–6. doi: 10.1177/0840470416679413 28929889 PMC5347358

[20] Clarke DE Dusome D Hughes L. Emergency department from the mental health client’s perspective. Int J Ment Health Nurs. (2007) 16:126–31. doi: 10.1111/j.1447-0349.2007.00455.x, PMID: 17348963

[21] Byrne SJ Bellairs-Walsh I Rice SM Bendall S Lamblin M Boubis E. A qualitative account of young people’s experiences seeking care from emergency departments for self-harm. Int J Environ Res Public Health. (2021) 18:2892. doi: 10.3390/ijerph18062892, PMID: 33808995 PMC8000083

[22] Saunders KE Hawton K Fortune S Farrell S. Attitudes and knowledge of clinical staff regarding people who self-harm: a systematic review. J Affect Disord. (2012) 139:205–16. doi: 10.1016/j.jad.2011.08.024, PMID: 21925740

[23] Taylor TL Hawton K Fortune S Kapur N. Attitudes towards clinical services among people who self-harm: systematic review. Br J Psychiatry. (2009) 194:104–10. doi: 10.1192/bjp.bp.107.046425, PMID: 19182168

[24] Frey LM Hans JD Cerel J. Perceptions of suicide stigma. Crisis. (2015) 37:95–103. doi: 10.1027/0227-5910/a000358 26695868

[25] Vedana KGG Magrini DF Miasso AI Zanetti ACG de Souza J Borges TL. Emergency nursing experiences in assisting people with suicidal behavior: a grounded theory study. Arch Psychiatr Nurs. (2017) 31:345–51. doi: 10.1016/j.apnu.2017.04.003, PMID: 28693869

[26] Bowden CF True G Cullen SW Pollock M Worsley D Ross AM. Treating pediatric and geriatric patients at risk of suicide in general emergency departments: perspectives from emergency department clinical leaders. Ann Emerg Med. (2021) 78:628–36. doi: 10.1016/j.annemergmed.2021.04.025, PMID: 34218952 PMC8546759

[27] O'Keeffe S Suzuki M Ryan M Hunter J McCabe R. Experiences of care for self-harm in the emergency department: comparison of the perspectives of patients, carers and practitioners. BJPsych Open. (2021) 7:e175. doi: 10.1192/bjo.2021.1006 PMC8935906 35264275

[28] Gibson R Carson J Houghton T. Stigma towards non-suicidal self-harm: evaluating a brief educational intervention. Br J Nurs. (2019) 28:307–12. doi: 10.12968/bjon.2019.28.5.307, PMID: 30907659

[29] O’Neill L Johnson J Mandela R. Reflective practice groups: are they useful for liaison psychiatry nurses working within the emergency department. Arch Psychiatr Nurs. (2019) 33:85–92. doi: 10.1016/j.apnu.2018.11.003, PMID: 30663630

[30] Michel K Gysin-Maillart A. ASSIP–attempted suicide short intervention program: a manual for clinicians. Göttingen, Germany: Hogrefe Publishing GmbH (2016).

[31] Self-harm and suicide in adults (CR229) Royal College of Psychiatrists. Available at: https://www.rcpsych.ac.uk/improving-care/campaigning-for-better-mental-health-policy/college-reports/2020-college-reports/cr229.

[32] Support for people who self-harm Samaritans Available at: https://www.samaritans.org/about-samaritans/research-policy/self-harm/.

[33] Vatne M Nåden D. Finally, it became too much–experiences and reflections in the aftermath of attempted suicide. Scand J Caring Sci. (2012) 26:304–12. doi: 10.1111/j.1471-6712.2011.00934.x, PMID: 22035225

[34] Betz ME Sullivan AF Manton AP Espinola JA Miller I Camargo CA Jr. Knowledge, attitudes, and practices of emergency department providers in the care of suicidal patients. Depress Anxiety. (2013) 30:1005–12. doi: 10.1002/da.22071, PMID: 23426881 PMC4350671

[35] Malaysia: religious affiliation: statista research Malaysia. Available at: https://www.statista.com/statistics/594657/religious-affiliation-in-malaysia/#statisticContainer.

[36] Rezaeian M. Islam and suicide: a short personal communication. Omega. (2009) 58:77–85. doi: 10.2190/OM.58.1.e 19112876

[37] Kelly BD. Self-immolation, suicide and self-harm in Buddhist and Western traditions. Transcult Psychiatry. (2011) 48:299–317. doi: 10.1177/1363461511402869, PMID: 21742954

[38] Potter J. Is suicide the unforgivable sin? Understanding suicide, stigma, and salvation through two Christian perspectives. Religions. (2021) 12:987. doi: 10.3390/rel12110987

[39] Foo XY Mohd Alwi MN Ismail SIF Ibrahim N Jamil Osman Z. Religious commitment, attitudes toward suicide, and suicidal behaviors among college students of different ethnic and religious groups in Malaysia. J Relig Health. (2014) 53:731–46. doi: 10.1007/s10943-012-9667-9, PMID: 23196328

[40] Kok JK van Schalkwyk GJ Chan AHW. Perceived stressors of suicide and potential prevention strategies for suicide among youths in Malaysia. Int J Sch Educ Psychol. (2015) 3:55–63. doi: 10.1080/21683603.2014.920285

[41] Tuti M Nursyuhaida M Khairunnisa M Marhani M Ruzanna Z. Stigma arising from family members of the mentally ill patients in hospital Taiping. Malays J Psychiatry. (2009) 18:13–22.

[42] Ritsher JB Otilingam PG Grajales M. Internalized stigma of mental illness: psychometric properties of a new measure. Psychiatry Res. (2003) 121:31–49. doi: 10.1016/j.psychres.2003.08.008, PMID: 14572622

[43] Corrigan PW Watson AC Barr L. The self–stigma of mental illness: implications for self–esteem and self–efficacy. J Soc Clin Psychol. (2006) 25:875–84. doi: 10.1521/jscp.2006.25.8.875

